# Uncooled two-microbolometer stack for long wavelength infrared detection

**DOI:** 10.1038/s41598-023-30328-1

**Published:** 2023-03-01

**Authors:** Amjed Abdullah, Akshay Koppula, Omar Alkorjia, Mahmoud Almasri

**Affiliations:** Electrical and Computer Engineering Department, University of Missouri Columbia, Columbia, MO USA

**Keywords:** Materials science, Optics and photonics

## Abstract

We have investigated an uncooled infrared (IR) detector utilizing a dual level architecture. This was achieved by combining two-microbolometer stack in the vertical direction to achieve high IR absorption over two distinct spectral windows across the long wavelength infrared region (LWIR). In addition, we have studied amorphous silicon germanium oxide (Si_x_Ge_y_O_1−x−y_) as an IR sensitive material, and metasurface to control IR absorption/reflection in interaction with standard Fabry–Perot cavity. The bottom microbolometer uses a metasurface to selectively absorbs a portion of the spectrum and reflects radiation outside this window range. At the same time, the top microbolometer uses a conventional Fabry–Perot resonant cavity to absorb a different portion of the spectrum and transmit any unabsorbed radiation outside this window. This device can be used to measure the absolute temperature of an object by comparing the relative signals in the two spectral bands. The spectral responsivity and detectivity, and thermal response time were > 10^5^ V/W, > 10^8^ cm Hz^1/2^/W, and 1.13 ms to filtered blackbody infrared radiation between (2–16) µm. The microbolometer voltage noise power spectral density was reduced by annealing the microbolometers in vacuum at 300 °C.

## Introduction

Uncooled multispectral thermal imaging IR cameras can be used in many applications such as defeat enemy camouflage, highlight battlefield hazards, chemical threats, mineral exploration, and agricultural inspection^1–3^. Thermal cameras that are based on single spectral window in the LWIR are used in many other commercial and military applications such as surveillance, threat detection, target recognition, security, medical diagnostics, and firefighting^4–7^. Many of these cameras are based on microbolometer technology, which is based on the changes of electrical resistance due to a change of temperature of the detector associated with the absorption of incident IR radiation. The changes of the electrical resistance of each microbolometer in the focal plane array (FPA) can be measured and converted into a thermal image. The majority of IR cameras are developed and manufactured by companies such as Raytheon, L-3 Communications, DRS, BAE Systems^8–11^, and many others^12–16^. These cameras are primarily based on two materials, vanadium oxide (VO_x_) and amorphous silicon (*a*:Si) technology with comparable detector performance. VO_x_ has TCR ranged from − 2 to − 2*.*4%/K^17^ while *a*:Si has TCR ranged from − 2.5 to − 5%/K. The corresponding resistivity ranges between 200 Ω-cm to 1 × 10^5^ Ω-cm^18^. The value of NETD and thermal response time for both detectors are < 50 mK, and < 10 ms, respectively, for a pixel size of 17 × 17 µm^2^ for spectral window in the LWIR^19^. Many other IR materials and detectors have been investigated such as Yttrium Barium Copper Oxide (YBaCuO)^20^, Si-Ge^21^, Si_x_Ge_y_O_1−x−y_^22–26^_,_ hydrogenated amorphous silicon (a-Si:H)^27,28^, yttrium (Y)-doped vanadium oxide (VOx:Y)^29^, graphene aerogel^30^, and various metals^31^.

Spectral selectivity has been implemented using a single FPA along with a filter wheel to select the appropriate spectral window^32,33^, two FPAs along with their electronics to operate in two spectral windows. The use of filter wheel or separate FPAs added complexity, cost, and the challenge of alignment between the FPAs^34^. The spectral selectivity can also be achieved using Fabry–Perot cavity where multiple pixels used in focal plane array (FPA) can be fabricated with different cavity heights to operate, at least in two spectral windows. However, this poses significant manufacturing challenge in terms of additional fabrication steps and several more photomasks. Several groups have experimented with dynamically tuning the microbolometer’s cavity, either through piezoelectric actuation^35^, electrostatic actuation^36,37^, liquid crystal-based changes in the refractive index^38^, or by using movable micromirror underneath the microbolometer pixel^39–41^. These approaches provide the ability to tune the wavelength response. However, the actuation requirements significantly complicate the fabrication, and reduce the resolution of the FPA.

An alternative to change the height of the cavity is to use metasurface, a class of perfect absorber, to control the spectral response. This approach has been recently explored using frequency selective surface (FSS) type of elements such as: asymmetric cross-shaped nanoparticle antennas^42^, multiplexed metamaterial structure in a single device or unit cell^43^, 2D absorber with Au dimples^44^, split ring resonators^45^, 1D and 3D metal–insulator-metal devices with a through-hole^46^, a metal cross pattern into a graphene layer^47^, plasmonic crystal absorbers^48^, and many others^49–52^. In one paper, the author patterned a number of square elements on the microbolometer pixel and showed the ability to tune the absorption in the MWIR^53^. A simpler metasurface hexagonal closed packed array patterned using microsphere photolithography technique was used to tune the resonance wavelength in the mid IR by changing the disk size while keeping the insulator attractive^54^. Square lattices were patterned on the microbolometer pixel to change the response of the microbolometer by changing the size of the post^55^. Other study performed experiments using FTIR, simulation where they put cross array on top of metasurface on top of *a*:Si, and planner multimode antenna based microbolometer^37,56^. They achieved narrow band across the LWIR^57^. In all these investigations, the metasurface was used as a paint to color the microbolometer. This painting approach added significant mass to the microbolometer, which will impact the microbolometer performance in terms of voltage responsivity and thermal response time.

The goal of this paper is to explore multi-level metasurface integrated geometry. This paper investigates a novel two-microbolometer stack architecture for detection of incident radiation in two distinct spectral windows in the LWIR region, a metasurface disk array patterned on the bottom microbolometer while a Fabry–Perot cavity was used as the top microbolometer, and an amorphous Silicon Germanium Oxygen (Si_x_Ge_y_O_1−x−y_) was selected as the IR sensitive material.

## Design and modeling of two-microbolometer stack

Metasurfaces can be used to control the way a structure interacts with radiation. While different paths to wavelength selectivity exist, these are generally characterized by using a filter to reject out of band radiation. This wastes the limited photons incident on the focal plan array (FPA) and reduces the sensitivity of the device. This device was designed with two- microbolometer stack to overcome this limitation, while maintaining a high fill factor, by splitting the radiation absorption between two microbolometers where each microbolometer captures a portion of the spectrum to maximize the IR absorptance over two distinct spectral windows in the LWIR, between 8 and 14 µm atmospheric transmission. The bottom microbolometer has a metasurface that selectively absorbs radiation between 8 and 11 µm, while reflecting radiation outside this range. The top structure is designed with a resonant Fabry–Perot cavity located between the top microbolometer pixel and the bottom metasurface to absorb incident radiation between 7.5 and 9 µm and transmit any unabsorbed radiation outside this window.

**Figure 1:**
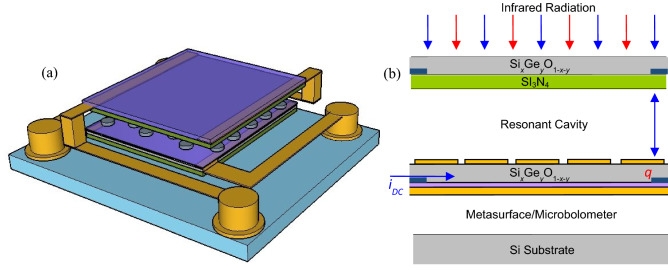
(**a**) 3-Dimensional of the two-microbolometer stack with a pixel area of 40 × 40 µm^2^ (**b**) schematic illustration showing the principal operation of the two-band microbolometer the metasurface design.

The two-microbolometer stack design as shown in Fig. 1a can be used to resolve the temperature of objects without a-priori knowledge of the object emittance. This approach will perform significantly better than a single-color sensor which estimates the temperature based on the radiant intensity and requires knowledge of the surface emissivity of an object to determine its temperature. The absorbed energy in each microbolometer raises the pixel’s temperature to be sensed. So, when incident radiation is absorbed by the metasurface in the bottom microbolometer it will be heated and conduct this energy to the sensing layer. Therefore, the bottom microbolometer is designed with metal–insulator-metal (MIM) configuration and suspended above the substrate, as shown in Fig. 1b, which allows selecting the desired resonant wavelength over the long wavelength region (LWIR). In this configuration, the Si_*x*_Ge_*y*_O_1−*x*−*y*_ semiconducting layer (300 nm) was encapsulated between the Aluminum (Al) ground plane (150 nm) and the metasurface, which is designed with a hexagonal close packed arrays of circular discs pattern, with a thin dielectric layer of SiO_2_ (50 nm) electrically isolating it from the ground plane. This is equivalent to a composite material such that the geometry determines the ensemble properties. The metasurface is arranged to be a perfect absorber over a narrow-band by matching a structure with a groundplane to free-space. The disc array is made by patterning Al with a thickness of 70 nm, and varied diameters (d) and periodicities (p) of 1.11 µm and 2.38 µm; 1.64 µm and 2.64 µm, respectively. In this configuration, the metasurface geometry control the absorbed wavelength by changing the diameter, periodicity, and height of the discs, where the resonant wavelength is strongly dependent on the disk diameter, and the magnitude of the absorptance depends on the impedance matching which can be achieved by adjusting the periodicity and height. We have used Al as a ground plane to achieve high reflectivity out of band. The Al ground plane prevents any transmission through the metasurface, so that any radiation that is not absorbed by the surface will be reflected. The bottom microbolometer, with pixel sizes of 40 × 40 µm^2^, consist of a thin Si_3_N_4_ bridge suspended above a silicon substrate and is supported by two narrow arms made of Si_3_N_4_ and NiCr (85/15). Encapsulated in the center of the Si_3_N_4_ bridge is the MIM structure. NiCr thin film was also patterned under Si–Ge–O layer to serve as an electrical contact. The two-microbolometer stack was modeled in HFSS (ANSYS) using a Floquet Port model (plane wave modal expansion) to simulate an infinitely periodic metasurface using a normally incident plane wave. The results were previously published in^58^.

The incident infrared radiation is partially transmitted through the top partially absorbing Fabry–Perot microbolometer pixel. The metasurface in the bottom microbolometer is reflective out of band and serves as the bottom surface of a Fabry–Perot cavity. Over this region, the top microbolometer acts the same way it would in a traditional microbolometer and with appropriate design, the resonant cavity maximizes the IR absorption in the microbolometer over this range. The resonant Fabry–Perot cavity can be created between the incoming and reflected waves if the cavity depth is tuned accordingly: *d*_*n*_ = [(2*n*−1)−(*φ*_1_−*φ*_2_)] *λ*/4, where *d*_*n*_ is the air gap depth, *n* is an integer, and *φ*_1_, and *φ*_2_ are the phase differences between the incoming and reflected light^59^, shown in Fig. 1b. An important portion of this is to adjust the impedance matching of the entire structure over the longer band while preserving absorptance in the top microbolometer in the shorter wavelength band. The top Fabry–Perot microbolometer, with pixel sizes of 40 × 40 µm^2^, consists of a thin Si–Ge–O bridge suspended above the metasurface. The bridge is supported by two narrow metallic arms of Si_3_N_4_ and NiCr. Encapsulated in the center of the Si_3_N_4_ bridge is a thin layer of Si_x_Ge_y_O_1−x−y_ IR sensitive material. The Si_3_N_4_ is used because of its excellent thermal properties, processing characteristics and good IR absorption.

The arms in both microbolometers serve as support structures, conductive legs and thermal isolation legs. They are designed with low thermal conductance while the pixels are designed with low thermal mass to reduce the thermal time constant and maximize the responsivity and detectivity. The thermal conductance through the electrode arms is made as small as possible with consideration of the response time requirements. NiCr was used as the electrical contact and electrode material as it makes excellent contact with Si–Ge–O resulting in linear behavior, and it has a relatively low thermal conductivity, providing better thermal isolation. A gold (Au) thin film was patterned under the polyimide sacrificial layer to define the traces and the bonding pads. The microbolometer was fabricated by sputtering the device layers on top of the polyimide sacrificial layer. Subsequent etching of the sacrificial layer provides the air gap that thermally isolates both microbolometers. An amorphous Silicon Germanium Oxygen (Si_x_Ge_y_O_1−x−y_) was selected as the IR sensitive material because it has a TCR higher than that of the mainstream IR materials that are currently used in the commercial thermal IR cameras such as VO_x_, while the resistivity could be reduced significantly without decreasing the TCR and is CMOS compatible which reduces fabrication expense. Amorphous Si_x_Ge_y_O_1−x−y_ films were co-sputtered from Si and Ge targets simultaneously from two deposition targets in an Ar/O_2_ environment at room temperature, low pressure 4 mTorr, and with a thickness around 300 nm. In addition, the voltage noise PSD was studied in detail with and without annealing in vacuum and in forming gases.

## Device fabrication

**Figure 2:**
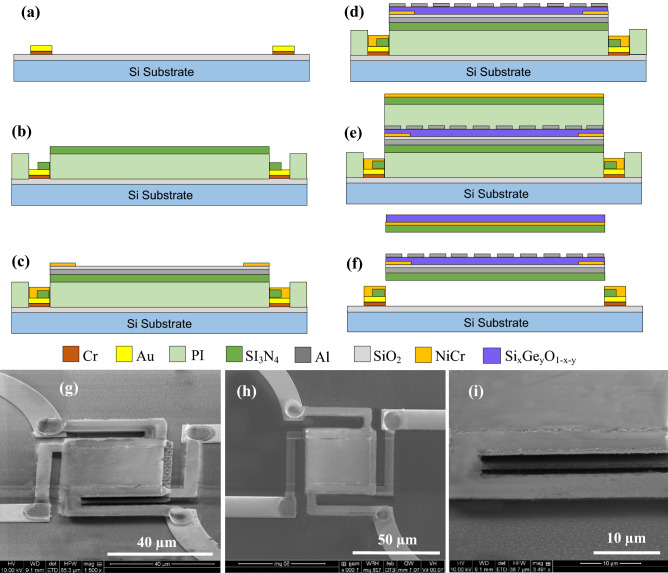
(**a**–**f**) Cross sectional view showing the fabrication steps of the two-microbolometer stack and (**g**–**i**) SEM micrographs of the fabricated two-microbolometer stack (left: tilted view, center: top view, and right: zoomed view of the cavities).

The microbolometer was fabricated using surface micromachining technology. The deposition of all layers was performed using RF magnetron sputtering equipped with two three-inch sputtering targets and co-deposition capabilities. Lift-off processes were used for patterning all microbolometer layers. Prior to the deposition, the sputtering chamber was evacuated to a base pressure of 0.5–3 µTorr. The chamber pressure during deposition was maintained at 4 mTorr by controlled Argon flow. The microbolometer was fabricated on a 3-inch oxidized silicon wafers as follow. (1) A negative photoresist (NR9-1000py) layer was patterned on an oxidized Si wafer at location corresponding to the lead lines and bonding pads for both microbolometers followed by sputter-deposition of a thin layers of Cr (60 nm) and Au (183 nm). The wafer then immersed in acetone with 3 min ultrasonic agitation to lift-off the unwanted Cr/Au layer forming the lead lines and bonding pads (Fig. 2a), (2) The air cavity was created using a polyimide sacrificial layer (PI2610), for thermally isolating the microbolometer from the substrate. It was spin coated, soft baked in oven at 140 °C for 40 min in N_2_ environment. The temperature was ramped up to 270 °C and the polyimide was cured for 2 h in N_2_ environment to obtain a layer with a thickness of 2.01 µm. The temperature was ramped down to under 100 °C before removing the wafer from the oven. A layer of photoresist with a thickness of 3.4 µm AZ4620 was spin coated and patterned, and the polyimide layer was then etched at location corresponding to the bottom microbolometer anchors, using Reactive Ion Etch (RIE) system. (3) Prior to depositing first Si_3_N_4_ bridge structure, a photoresist layer of NR9-1000py was patterned followed by sputter deposition and patterning, using lift-off technique, of a layer of Si_3_N_4_ with a thickness of 391 nm (Fig. 2b). This is followed by sputtering and patterning a layer of Al (169 nm) to form the ground plane, and a layer of SiO_2_ (43 nm) to isolate the subsequent layer of Si–Ge–O from the ground plane. (4) The electrode and electrical contacts were formed by sputtering and patterning a layer of NiCr (80% Ni and 20% Cr) with a thickness of 83 nm, using liftoff process (Fig. 2c). (5) The Si_x_Ge_y_O_1−x−y_ films were co-sputtered from Si and Ge targets simultaneously from two deposition targets in an Ar/O_2_ environment at room temperature, low pressure 4 mTorr. The films were then patterned with a thickness around 293 nm, using lift-off process. (6) The Al metasurface layer was then deposited and patterned with circular disk arrays with a varying diameter (d) and periodicity (p). The thickness was 74 nm, again, it was patterned using lift-off process (Fig. 2d). (7) The top conventional microbolometer was started by spin coating and curing of the polyimide (PI2610) sacrificial layer of 2.01 µm thickness to isolate the top microbolometer from the bottom one, provide thermal isolation, and create resonant cavity. (8) The bridge structure for the top microbolometer was created by pattering NR9-1000 py photoresist and sputter deposition of Si_3_N_4_ layer with a thickness of 291 nm. (9) This was followed by deposition and patterning the NiCr electrode and electrical contacts with a thickness of 83 nm using NR9-1000 photoresist and lift-off process (Fig. 2e). (10) The second Si–Ge–O sensing layer was sputter deposited for the top microbolometer with a thickness of 292 nm. (11) In the final step, two stack microbolometer was suspended by removing the polyimide sacrificial layer using plasma ashing system, under oxygen environment and 350 mT (Fig. 2f). The SEM micrographs of the fabricated devices are shown in (Fig. 2g–i).

## Testing and characterization

### Resistance versus temperature characteristics

**Figure 3:**
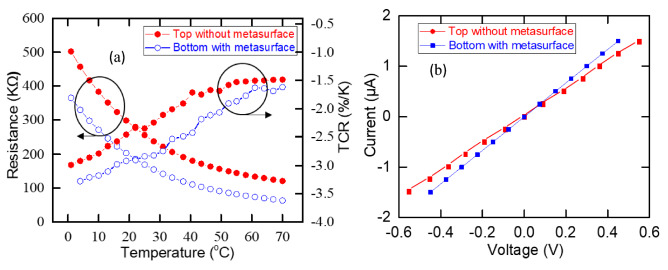
(**a**) TCR of the fabricated devices, and (**b**) Current–voltage characteristics of the fabricated device.

The resistance versus temperature (R–T), and temperature coefficient of resistance (TCR), also known as $\beta$, were measured. TCR defined as a quantifier that tells how rapidly the resistance of a material responds to a change in temperature. It is determined by

1 $$= \frac{1}{R}\frac{dR}{{dT}} = \frac{1}{R}\frac{\Delta R}{{\Delta T}} = - \frac{{E_{a} }}{{K_{B} T^{2} }}$$

where ${E}_{a}$ is the activation energy, ${K}_{B}$ is the Boltzmann constant, and T is the absolute temperature. The measurement was performed by mounting the fabricated devices inside Janis VPF-100 cryostat, onto a stage designed for 4-point probe measurement. The cryostat was evacuated to 30 mT. A programmable current source (Keithley Model 220) and a high precision voltmeter (Keithley model 2182 nano-voltmeter) were used to apply a fixed current across the outer two points and measure the resulting voltage across the inner two points in the 4-probe setup. A Lakeshore 336 controller was used to vary the temperature from 0 to 70 °C with 2 °C intervals. 150 data points were measured and averaged at each temperature set-point to study the R–T characteristics. The measured TCR at room temperature (295 K) for the bottom microbolometer with metasurface was − 3.01%/K while it was − 2.34%/K for the top microbolometer (without metasurface) as plotted in Fig. 3a. The bottom microbolometer showed higher TCR due to the presence of the metasurface. The current–voltage characteristics were measured after annealing the devices and plotted in Fig. 3b. The results demonstrated that the current voltage relationship is linear up to 1.5 µA. Joule heating was observed only above 1.5 µA. The typical two-wire resistance of the devices were ranged from 78 to 639 k.

### Noise testing

**Figure 4:**
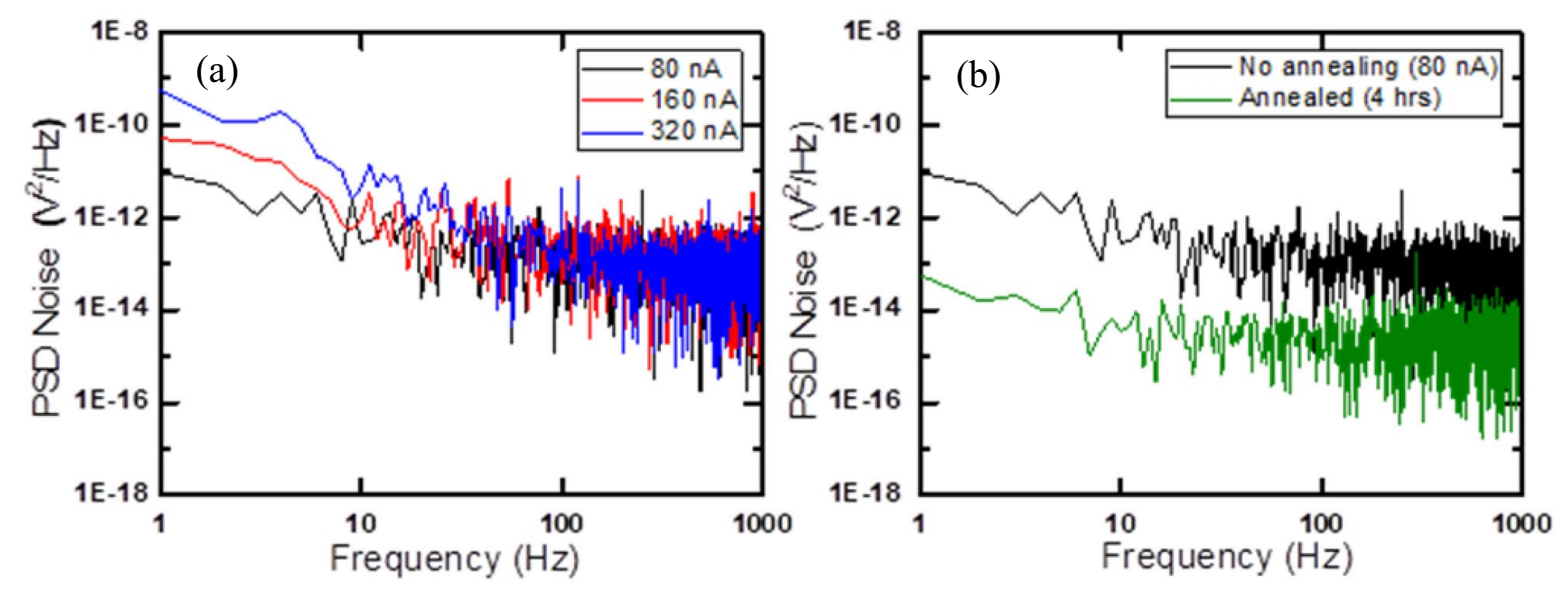
Voltage noise PSD of the bottom microbolometer (**a**) before annealing for three different biased current from 80 to 320 nA (**b**) annealing in vacuum for 4 h at 80 nA.

The noise voltage power spectral density (PSD) of the two-microbolometer stack with a pixel area of 40 × 40 µm^2^ was measured and reduced by annealing the devices in vacuum and at 300 °C for 4 h duration. The devices were measured before and after annealing at different bias currents. The measurements were performed in air inside a cryostat (DE 202 cold head), inside an EM shielded box in order to reduce any external noise such as 60 Hz noise. The microbolometer was connected to a 1 MΩ metal resistor voltage biased using Nickel–Cadmium (Ni–Cd) battery that generates current ranged from 80 to 320 nA. Figure 4a shows the noise PSD spectrum before annealing for the bottom microbolometer with metasurface. The figure clearly demonstrates that the noise increases as the biasing current increase. The lowest measured voltage noise PSD at the corner frequency 90 Hz, where Johnson noise meet 1/*f-*noise, was 1.13 × 10^–13^ V^2^/Hz for the device that was biased with 80 nA.

The devices were annealed in vacuum at 300 °C for 4 times intervals. Each time interval lasted for 1 h. The vacuum annealing was performed inside a Rapid Thermal Annealing (RTA) system (MILA Mini Lamp Annealer) at a base pressure of 1.5 mTorr. The temperature was ramped up to 300 °C at a rate of 25 °C/min. The annealing process passivate the defects at the grain boundaries. The voltage noise of the bottom microbolometer (with metasurface) was measured after vacuum annealing as shown in Fig. 4b. The results demonstrate that the voltage noise was reduced with the lowest measured voltage noise PSD at the corner frequency 8 Hz was 3.41 × 10^–15^ V^2^/Hz for the device that was biased with 80 nA. The voltage noise before annealing at the same bias current and corner frequency was 8.51 × 10^–12^ V^2^/Hz at 8 Hz. This is 2.49 × 10^3^ times reduction in noise. The 1/*f*-noise can be determined using Hooge’s empirical Eq. ^60^.

2 $$S_{\frac{1}{f}} = \frac{{K_{f} \left( {I_{B} R} \right)^{\beta } }}{{f^{\gamma } }}$$

where S_1/*f*_ is the noise voltage PSD, *f*_γ_ is the electrical frequency with γ close to 1 for 1/*f-*noise, K_f_ is the 1/*f*-noise coefficient (flicker noise coefficient). It depends on the quality of the crystal, and on the scattering mechanisms that determine the mobility μ. I_B_ is the bias current and R is the resistance of the device. The product I_B_ × R is the applied DC bias voltage (V_dc_), and the ideal value of β is 2 which indicates that doubling the bias current will double the voltage noise at low frequency noise. The values of γ, β, and *K*_*f*_ were determined by taking the logarithm of Eq. (2).

3 $$log_{10} \left( {S_{\frac{1}{f}} } \right) = \beta log_{10} \left( {I_{B} } \right) - \gamma log_{10} \left( f \right) + log_{10} \left( {K_{f} } \right) + \beta log_{10} \left( R \right)$$

The straight-line equation has a slope equals *β*, and the last two terms are constants at a specific frequency. To calculate the value of *β* we plotted log_10_(S_1/*f*_) as a function of log_10_(I_B_) using frequency range between 1 and 10 Hz for 3 different bias currents. We first determined the slope (*β*) for each bias current and then calculated the average value of *β* from the three slopes. Similarly, the value *γ* was calculated from the average slope of the voltage noise versus currents plot for the same 3 different bias currents in the frequency range 1–10 Hz. The average value of *K*_*f*_ was determined by substituting the calculated value of *γ* and *β* for each bias current into Eq. (7) at 10 Hz. The corresponding Hooge’s parameters γ, β and *K*_*f*_ for the bottom microbolometer device before and after annealing in vacuum were 1.31, 1.88, 3.81 × 10^−12^, and 1.11, 1.93, 6.92 × 10^−13^, respectively. The value of γ is close to 1 after annealing which indicates that the 1/*f-*noise is dominant at low frequencies. The decrease in *K*_*f*_ after annealing is attributed to the reduction of 1/*f*-noise. The *K*_*f*_ value depends on the quality of the crystal, and on the scattering mechanisms that determine the mobility *µ*^61^. The β value was close to 2 when the device was annealed for 4 h.

The voltage noise results before annealing the devices demonstrate that the presence of defects in the amorphous Si_x_Ge_y_O_1−x−y_ sensing layer, trapping and de-trapping of electrons between the conduction band and the traps, and the cleanliness of the Ni–Cr metal contacts with the Si_x_Ge_y_O_1−x−y_ semiconductor interface are the main sources of the large 1/*f-*noise. The Ni–Cr and Si_x_Ge_y_O_1−x−y_ interface may have formed Schottky junction that have increased the noise. The presence of large number of defects in the sensing layer are mainly due to bond formation of the Si_x_Ge_y_O_1−x−y_ sensing layer in terms of Si–O and Ge–O, may be due to non-equilibrium state from the glow discharge in the sputtering process^25^. These bonds have resulted in large number of defects in the film and contributed to the presence of 1/*f*-noise. In the same manner, Ge–O and Ge–Ge bonding leads to dangling bond of Ge. They occur in variety of situations: at interfaces, on surfaces, and in point defects such as vacancies. Causing defects in the sensing layer and thus increases the voltage noise level. In addition, due to the presence of large number of localized states in the amorphous semiconductors, in our case the Si_x_Ge_y_O_1−x−y_ sensing layer, a generation recombination noise is used to explain the conductance fluctuations. The localized state traps restrict the movement of electrons and holes in which reduces the conductance and thus produce a Lorentzian noise spectrum. The SiGe trap centers alloy is located close to the midgap, and the presence of these traps tend to contribute to the noise spectrum at lower frequencies^62,63^.

The voltage noise was reduced significantly at 4 h annealing in vacuum, which indicates that the vacuum annealing played a big role in reducing defects and trapping states, and fixing the dangling bond, recombination centers, and improving the metal semiconductor interface. After 4 h of annealing, the voltage noise PSD including 1/*f-*noise started to increase. This is probably due to the increase of dangling bonds, low field mobility, and the oxide traps^64,65^.

### Optical Measurements

The optical response of the two-microbolometer stack was measured as a function of wavelength and as a function of chopping frequency. The voltage Responsivity is defined as the output voltage signal divided by the incoming input radiation power with respect to the pixel area. It is given by^66^.

4 $$Rv = \frac{{I_{b} R\beta \eta }}{{G\left( {1 + \omega^{2} {\mathcal{T}}_{th}^{2} } \right)^{1/2} }}$$

where $I_{b}$ is the bias current, R is the device resistance, $\eta$ is the absorptivity, $\omega$ is the radiation modulation frequency, ${\mathcal{T}}_{th}$ is the thermal response time, defined by the ratio of the device thermal mass to the thermal conductance. It is a tradeoff between speed and sensitivity. The voltage responsivity is scaled up by the bias current, TCR, absorptivity, resistance, and normalized with respect to thermal conductance. The voltage detectivity represents the signal-to-noise ratio (SNR) normalized with respect to the detector’s pixel area.

5 $$D* = \frac{{Rv\sqrt {\Delta f A} }}{{\Delta V_{n} }}$$

where $\Delta f$ is the frequency bandwidth read from the dynamic signal analyzer. $\Delta {V}_{n}$ is the noise voltage, including the background noise, the temperature fluctuation noise, and the noise generated by the thermometer, which is made up by the Johnson noise and the 1/*f*-noise.

**Figure 5:**
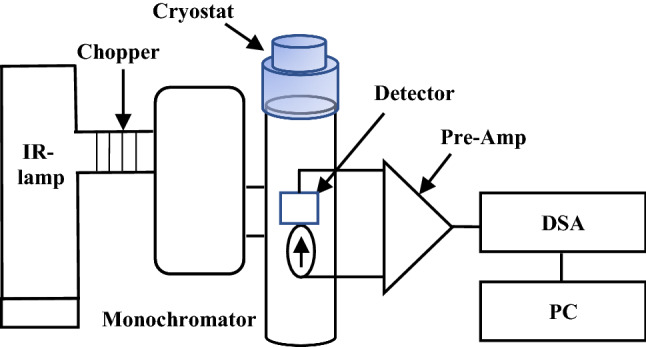
A schematic showing the spectral Responsivity and Detectivity testing setup.

**Figure 6:**
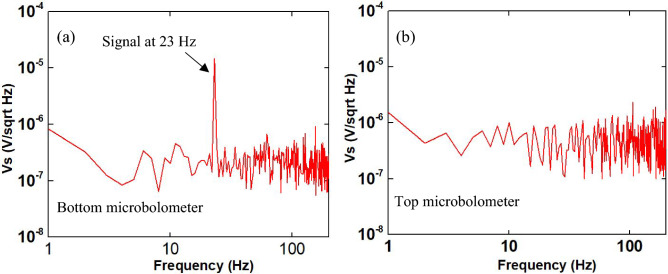
A typical voltage spectrum measured in vacuum at a bias current of 320 nA in response to a Blackbody light source chopped at 23 Hz at and wavelength of 10.3 µm illuminated the detector with d = 1.64, p = 2.64.

A schematic diagram of the testing setups is shown in Fig. 5. It includes blackbody light source (Newport 60,090), an infrared ceramic element Newport 6575) with radiation wavelength between 0.6 and 16 µm, a ZnSe condenser (Newport 60,077), a monochromator (Newport 77,100), and an optical long pass filter with transmittance between 2 and 16 µm. The measurements were performed in vacuum inside a cryostat through a ZnSe window (2–16 µm), and inside an EM shielded box. The microbolometer was mounted on a vertical stage inside the cryostat allowing the chopped light, that is controlled by a chopper frequency controller to fully illuminate the microbolometer surface through the ZnSe window. The devices were connected to a preamplifier (PAR5113) and a Hewlett-Packard 35670A dynamic signal analyzer, and were DC biased from 80 to 320 nA. The dynamic signal analyzer simultaneously measured the signal amplitude and noise per unit bandwidth for each chopper frequency. The spectral responsivity and detectivity of fabricated microbolometers were also measured in vacuum at room temperature. The signal and noise were recorded for each wavelength between 4  and 16 µm while the light was chopped at 23 Hz. The response was calibrated at each chopping frequency and at each wavelength with a Newport pyroelectric detector (70,363) with known Responsivity and area. The spectral response of the microbolometer was measured in vacuum as a function of wavelength over the range of 4–16 µm. Figure 6 shows a typical voltage spectrum in vacuum at a bias current of 80 nA in response to a Blackbody radiation chopped at 23 Hz and the light with a wavelength of 9 µm illuminated the microbolometer. The bottom microbolometer shows strong signal at 23 Hz while the top microbolometer shows weak signal.

**Figure 7:**
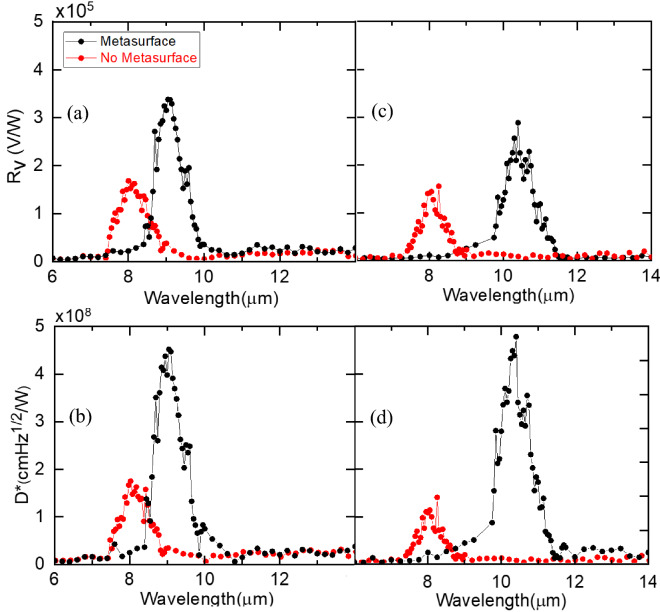
Measured responsivity and detectivity as a function of wavelength at 320 nA bias current for (**a**, **b**) detector with d = 1.11, p = 2.38 and (c, d) detector with d = 1.64, p = 2.64.

The spectral response of each microbolometer in the two-microbolometer stack was measured and shown in Fig. 7. The measurement demonstrates that the spectral response changes as a function of metasurface. Narrow band was achieved at certain wavelength depending on the geometry of the metasurface. When the disk diameter and periodicity changed, the spectral response was shifted to a different spectral window. Also, each microbolometer captures a portion of the spectral window with the top microbolometer response peak at 8 µm. These results demonstrate the ability of metasurface to tune the spectral response and multispectral absorption by stacking two microbolometers on a single pixel size.

**Figure 8:**
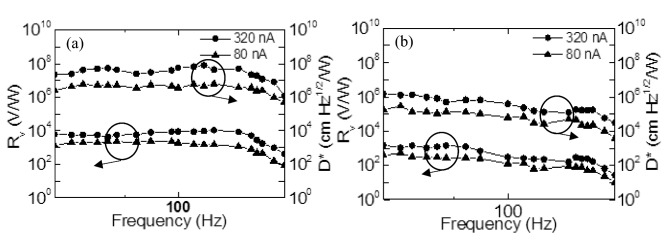
Measured voltage responsivity and detectivity as a function of chopper frequency at different bias currents for (**a**) bottom microbolometer (d = 1.11, p = 2.38), and (**b**) top microbolometer.

The voltage responsivity as a function of chopper frequency was also measured for the bottom and top microbolometers. In this case, the monochromator was removed from the testing setup and the chopper was placed very close to the microbolometer. The results were plotted as a function of chopper frequency at different bias currents in Fig. 8. The bottom microbolometer with metasurface showed better responsivity and detectivity due to the inclusion of the metasurfaces with the sensing layer which improved the response of the microbolometer and increased the sensitivity.

The thermal conductance G_th_ of the microbolometer is measured by Joule heating method at different temperature and calculated by the following Eq.^67^.

6 $$R\left(T\right)={R}_{0}+\frac{1}{{G}_{th}}\frac{dR}{dT} {I}_{b}^{2}R\left(T\right)$$

The thermal conductance was calculated from the TCR and the slope of the device resistance versus the dissipated power curve at room temperature for the bottom and top microbolometer to be 7.78 × 10^–8^ W/K and 5.24 × 10^–8^ W/K, respectively. The thermal response time was calculated from the cut off frequency to be 1.13 ms. The thermal mass was calculated from the measured thermal conductance and thermal response time to be as 2.26 × 10^–10^ J/K. The absorptivity was calculated to be 37.54% and 21.15% by fitting the measured data for responsivity, thermal conductance, and thermal response time. A summary of the two-microbolometer stack results is presented in Table 1.

**Table 1:** Summary of the metasurface enabled microbolometer properties.

Description	Bottom microbolometer/FSS	Top microbolometer
Pixel area (µm^2^)	40 × 40	40 × 40
Responsivity ${R}_{v}$(V/W)	1.07 × 10^4^	2.69 × 10^3^
Detectivity D* (cm-Hz^1/2^/W)	7.98 × 10^7^	1.52 × 10^6^
Thermal Conductance G_th_ (W/K)	8.55 × 10^–8^	2.94 × 10^–8^
Response time ʈ (ms)	1.18	3.32
Absorptivity $\zeta$	46.4	21.97
Thermal mass C (J/K)	1.01 × 10^–10^	9.75 × 10^–11^

## Conclusion

This paper presents the design, fabrication, and testing of 40 × 40 µm^2^ uncooled two-microbolometer stack, fabricated on top of each other. Each microbolometer captures a portion of the spectrum to maximize the IR absorptance over two distinct spectral windows in the LWIR. The bottom microbolometer was fabricated with a metasurface to selectively absorb radiation between, e.g., *λ* = 8–12 µm, while reflecting radiation outside this range. The top microbolometer was fabricated with a resonant Fabry–Perot cavity located between the top microbolometer pixel and the bottom metasurface in order to absorb incident radiation between 7.5 and 9 µm and transmit any unabsorbed radiation outside this window. The two-band design can be used to measure the absolute temperature of an object by comparing the relative signals in the two spectral bands. The spectral responsivity and detectivity, thermal response time, thermal conductance, and absorptivity of the bottom and top microbolometers were 3.4 × 10^5^ V/W and 1.7 × 10^5^ V/W, 4.3 × 10^8^ cm Hz^1/2^/W and 2.1 × 10^8^ cm Hz^1/2^/W, 1.1 ms and 3.79 ms, 6.55 × 10^–8^ W/K and 4.34 × 10^–8^ W/K, 34.74% and 21.51%, respectively. The voltage noise PSD was lowered using vacuum annealing and the corner frequency was shifted to a lower value.

## Data Availability

The datasets used and/or analyzed during the current study are available from the corresponding author on reasonable request.
